# CRHunter: integrating multifaceted information to predict catalytic residues in enzymes

**DOI:** 10.1038/srep34044

**Published:** 2016-09-26

**Authors:** Jun Sun, Jia Wang, Dan Xiong, Jian Hu, Rong Liu

**Affiliations:** 1 Hubei Key Laboratory of Agricultural Bioinformatics, College of Informatics, Huazhong Agricultural University, Wuhan 430070, P. R. China

## Abstract

A variety of algorithms have been developed for catalytic residue prediction based on either feature- or template-based methodology. However, no studies have systematically compared these two strategies and further considered whether their combination could improve the prediction performance. Herein, we developed an integrative algorithm named CRHunter by simultaneously using the complementarity between feature- and template-based methodologies and that between structural and sequence information. Several novel structural features were generated by the Delaunay triangulation and Laplacian transformation of enzyme structures. Combining these features with traditional descriptors, we invented two support vector machine feature predictors based on both structural and sequence information. Furthermore, we established two template predictors using structure and profile alignments. Evaluated on datasets with different levels of homology, our feature predictors achieve relatively stable performance, whereas our template predictors yield poor results when the homological relationships become weak. Nevertheless, the hybrid algorithm CRHunter consistently achieves optimal performance among all our predictors. We also illustrate that our methodology can be applied to the predicted structures of enzymes. Compared with state-of-the-art methods, CRHunter yields comparable or better performance on various datasets. Finally, the application of this algorithm to structural genomics targets sheds light on solved protein structures with unknown functions.

Enzymes play indispensable roles in catalyzing almost all biochemical reactions in living organisms. Key residues that are directly involved in various catalytic processes are typically defined as catalytic residues. The identification of these functionally critical residues not only deepens our understanding of the catalytic mechanisms of enzymes but also offers valuable insights that can be used in enzyme engineering and drug discovery. Given the rapid increase in the number of protein sequences and structures, efficient ways to address this issue are urgently required. The experimental validation of catalytic residues is time-consuming and labor-intensive. Therefore, the computational prediction of these residues might complement the shortcomings of experimental methods.

To date, intensive efforts have been made to develop a variety of algorithms for catalytic residue prediction. Homology- or template-based strategies might be the most straightforward way to solve this problem. The putative catalytic residues of a query protein can be inferred by comparing its sequence or structure with the homologous proteins containing experimentally validated residues. For example, Wallace *et al*.^1^ first developed a program termed TESS, which used a geometric hashing algorithm to align novel protein structures against the structural template database of various enzyme active sites. Nebel *et al*.^2^ also implemented a structure-based method to discover the active sites of protein with rigid prosthetic groups based on clues from multiple templates. At the sequence level, Mistry *et al*.^3^ proposed a group of strict rules that enable the transfer of previously verified catalytic residues to other chains within the same Pfam family. Unfortunately, these studies did not systematically investigate the extent to which sequence or structural similarity can result in the reliable transfer of information regarding catalytic residues. Additionally, the greatest weakness of the above approaches is that they strongly depend on the availability of reasonable templates, thus enabling a relatively narrow range of applications.

In contrast, the vast majority of current algorithms utilized feature-based strategies as alternative ways to predict catalytic residues. To this end, previous studies have proposed a large number of sequence and structural features that can be effectively used to characterize these critical residues. For instance, catalytic residues prefer higher sequence conservation^4^ and closeness centrality^5^, lower solvent accessibility and flexibility^6^,^7^, and pocket or cleft regions in enzyme structures^6^,^8^,^9^. Based on the above attributes, a large number of feature-based algorithms have been established using customized scoring functions or machine learning techniques as their prediction engines. For instance, Lichtarge *et al*.^10^ designed an evolutionary trace method that considered the most highly conserved residues to be involved in catalytic processes. Gutteridge *et al*.^11^ constructed a neural network-based predictor to identify catalytic residues based on structural information. Petrova and Wu^12^ and Youn *et al*.^13^ developed support vector machine-based approaches using a variety of sequence and structural properties. Zhang *et al*.^14^ also used a support vector machine to implement their algorithm, but established a pure sequence-based predictor. Xin *et al*.^15^ developed a structure kernel method by incorporating multiple features. The aforementioned feature-based approaches also represent the state-of-the-art in catalytic residue prediction. Despite the great progress achieved by previous studies, well-designed feature-based algorithms remain highly anticipated, especially those incorporating novel characteristics. To our best knowledge, further, none of the existing studies systematically compared the strengths and weaknesses of feature- and template-based strategies or considered whether their combination could be used to improve catalytic residue prediction.

In this work, we attempted to simultaneously exploit the complementarity between feature- and template-based methodologies and that between structural and sequence information for catalytic residue prediction. First, Delaunay triangulation and Laplacian transformation were used to characterize enzyme structures, thereby generating several novel structural attributes. By integrating these features with traditional descriptors, we established two support vector machine feature predictors based on structural and sequence information. We also developed two template predictors using structure and profile alignments, respectively. An integrative prediction algorithm termed CRHunter was finally generated to recognize catalytic residues by combining the above four predictors. When evaluating our approach in different scenarios, we assessed the strengths and limitations of our different predictors. Overall, CRHunter not only achieves improvements over both its structure- and sequence-based component predictors but also outperforms the state-of-the-art algorithms. The final application to structural genomics targets further highlights the usefulness of our algorithm. The CRHunter server is freely available at http://www.bioinfo-hzau.cc/CRHunter/.

## Materials and Methods

### Overview of our prediction system

**Figure 1:**
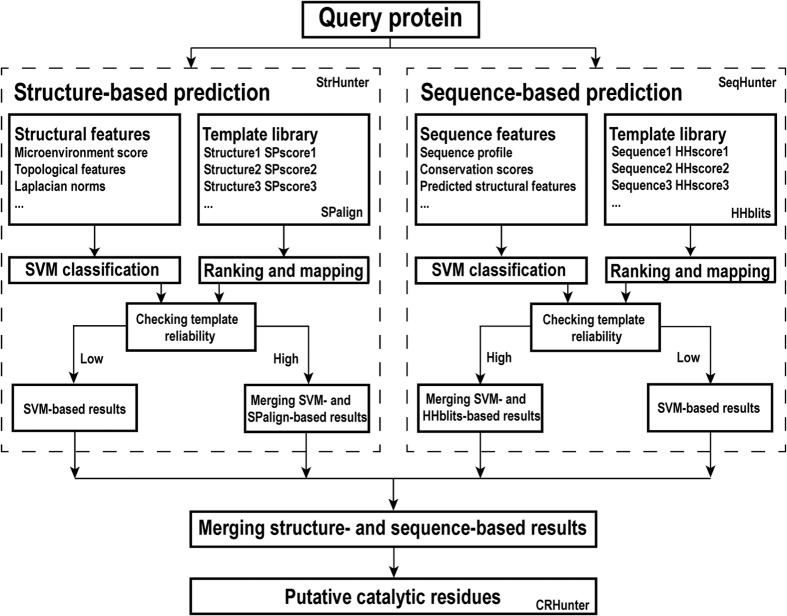
Schematic representation of the CRHunter algorithm. CRHunter is divided into two partitions, namely StrHunter and SeqHunter, both of which further comprise feature- and template-based predictors.

As shown in Fig. 1, the proposed system is separated into two partitions, namely structure- and sequence-based prediction modules, which further comprise feature- and template-based predictors, respectively. Regarding structure-based prediction, our template method can locate potential catalytic residues based on the global structural similarity between the query enzyme and its well-aligned reference structures, and our feature method provides complementary signatures by combining machine learning techniques and local structural characterization. In contrast, because protein structures have not been solved for all enzymes, we extended the integrative strategy of our structure-based module to sequence-based prediction. Therefore, effective sequence characteristics were extracted as the inputs of the other feature predictor, and structure alignment was replaced by sequence profile alignment in the template predictor. These four component predictors were integrated to establish our ultimate prediction algorithm.

### Feature generation

We defined several novel structural descriptors based on the Delaunay triangulation and Laplacian characterization of enzyme structures as described in the following sections. To our knowledge, these features have been applied to catalytic residue prediction for the first time. Moreover, we extracted traditional structural and sequence features for each residue, such as solvent accessibility, pocket information, position-specific scoring matrix (PSSM), and predicted structural features (see Supplementary Methods).

#### Generation of residue microenvironment using Delaunay triangulation

**Figure 2:**
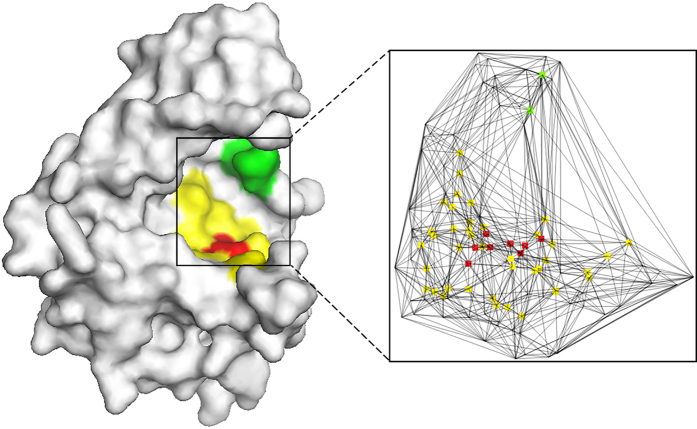
Generation of the microenvironment for each residue using Delaunay triangulation. In the left image, the catalytic residue of an enzyme structure (SCOP ID: d1gpma1) is shown in red, and its neighboring residues are shown in yellow or green. The right image shows the Delaunay triangulation of this active site, in which each node represents an atom. The atoms of the catalytic residue are shown in red, and atoms of neighbors that share a common facet with any atom of the catalytic residue are shown in yellow or green. Residues shown in green are finally removed because the number of common facets is less than the optimal cutoff.

In this study, we used the Delaunay triangulation (DT) to generate the microenvironment of each residue^16^. For each target protein, the qdelaunay application of the Qhull package was used to divide the three-dimensional structure into tetrahedrons such that each vertex represents an atom^17^. We also generated a Voronoi diagram for atoms in the target protein, which corresponds to the geometric dual of Delaunay triangulation. In this context, two residues are considered to be in contact if any pair of heavy atoms from each residue shares a common facet in the Voronoi diagram. Figure 2 shows an example of the DT-based microenvironment of a catalytic residue.

#### Microenvironment score based on Delaunay triangulation

Using the above neighborhood, we defined a feature termed MEscore_DT_ inspired by the work of Han *et al*.^18^, which utilized the preference of specific residue pairs in the local environment to identify catalytic residues. We first constructed the residue pair frequency vector (termed *F*_*DT*_) for each residue as follows:


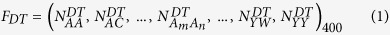


where *A*_*m*_ and *A*_*n*_ refer to the query residue and its neighbor, respectively, and 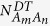 denotes the number of this residue pair in the DT-based microenvironment. We then calculated the residue pair weight vector of each catalytic residue as follows:


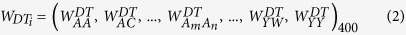



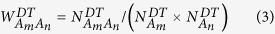


where 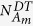 and 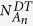 denote the counts of *A*_*m*_ and *A*_*n*_ in the DT-based neighborhood. We further computed the overall *W*_*DT*_ by averaging all of the weight vectors of catalytic residues in the training set. Finally, MEscore_DT_ was defined as follows:


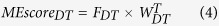


where 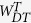 is the transposed matrix of *W*_*DT*_.

Because the number of common Voronoi facets of a given residue pair can reflect its contact strength, we deleted neighbors having a weak interaction with the target residue (Fig. 2). When MEscore_DT_ is individually used for prediction with optimal performance, the number of common facets should be no less than 9 (see Supplementary Fig. S1).

#### Topological features based on Delaunay triangulation

Using the DT-based neighborhood, we transformed each enzyme structure into a residue interaction network in which vertices represent residues and edges denote linkages between residues. Two residues are considered to form a linkage when they share at least 9 common facets. For each residue, we computed four network parameters in which closeness and betweenness assess its global importance in the network, degree represents its local connectivity, and clustering coefficient reflects the compactness of its neighboring residues.

#### Geometric features based on Laplacian characterization

The Laplacian characterization of protein structures has recently been applied to structural comparison^19^ and RNA-binding residue prediction^20^. Here we extended the application of this method. Briefly, we computed a group of features termed Laplacian norms (LNs), which measure the distance between a target residue and the weighted center of its neighboring residues on multiple scales. The alpha carbon was utilized to represent each residue. Then a discrete Laplace operator was calculated as follows:


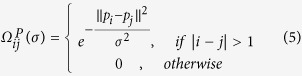


where *p*_*i*_ and *p*_*j*_ denote the Cartesian coordinates of residue *i* and its neighbor *j*, respectively. The parameter *σ* is a scale factor that reflects the weights of close and remote neighbors. A lower value of *σ* indicates that only the nearby residues make an important contribution to the computation, whereas a higher value of *σ* indicates that the distant residues are also useful. To obtain this parameter, we calculated the distance distribution of all residue pairs in each protein. The values at the 0, 2^−6^, 2^−4^, 2^−2^, and 1 quantile positions of this distribution were chosen as the scale factors by systematical sampling (see Supplementary Fig. S2). The norm of the Laplacian coordinates of the target residue was then generated as follows:


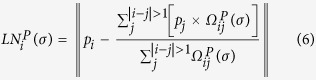


As a result, the LN features of each residue are represented by five weighted distances based on the selected scale factors.

### Feature-based predictors

After generating the above features, we established two support vector machine (SVM)^21^ feature predictors using structural and sequence information. For our structural predictor, we utilized all structural descriptors (see Supplementary Methods) and the sequence profile in combination with the structural neighborhood as its inputs. Neighboring residues in the DT-based microenvironment were sorted in terms of the number of facets shared by these residues and the target residue. For our sequence predictor, we input all sequence features (see Supplementary Methods) combined with the sequence microenvironment, which is a sliding window centered on the target residue. These predictors were implemented using LIBSVM^22^. The ratio of catalytic to non-catalytic residues in the training set was assigned as 1:6. To scale the attribute values to [0, 1], the elements in PSSM were transformed using the logistic function, and all remaining features except the binary vectors were transformed into a Z-score followed by the logistic function. By testing different window sizes, we found optimal values of 4 and 9 for the structure- and sequence-based predictors, respectively. To determine the parameters of the radial basis function in LIBSVM, we conducted a grid search for each predictor in which both C and γ values were tested in the range from 2^−5^ to 2^5^, with a step size of 2. When these two parameters were set to 2 and 0.03125, respectively, both feature predictors achieved optimal performance.

### Template-based predictors

We further developed two template predictors based on structure and sequence alignments. For the first predictor, we compared the query structure against each enzyme in the template library using SPalign^23^. Based on the structural similarity score (SPscore), the top-ranked enzyme was used as the reference structure and its catalytic residue information was transferred to the query structure. Concretely, if one residue in the query was matched with a catalytic residue in the optimal template, this position was considered as a catalytic residue with a probability score of 1; otherwise, a probability score of 0 was assigned. For the second predictor, the HHblits program^24^, a profile hidden Markov model (HMM)-based algorithm, was used as our search engine. We converted both the query and template sequences into HMMs by searching the sequences against the Uniprot20 database and conducted profile-profile alignments using the query HMMs and the template HMMs library. This predictor selected the best template based on the profile similarity score (HHscore) and mapped known catalytic residues to the query sequence as with the structural template method.

### CRHunter: integrating the above four predictors

Our prediction system is an ensemble algorithm integrating the complementarity between feature- and template-based methods and that between structural and sequence information. Template-based methods might provide higher prediction accuracy but unfortunately lose their power in the absence of good templates. Contrarily, coupled with higher false positive rates, feature-based methods might have broader applications^25^,^26^,^27^. Considering these points, we combined the structural feature- and template-based predictors as follows:


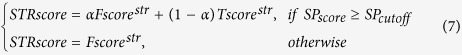


where *Fscore*^*str*^ and *Tscore*^*str*^ are the outputs of the individual predictors. The values of weight *α* and structural similarity cutoff *SP*_*cutoff*_ were 0.55 and 0.6, respectively. Likewise, we merged the two sequence-based predictors as follows:


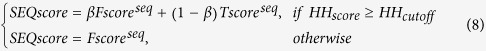


where the values of weight *β* and profile similarity cutoff *HH*_*cutoff*_ were 0.57 and 0.87, respectively. For the structure- and sequence-based modules, we further combined their outputs as follows:


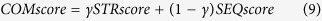


where weight *γ* was 0.5. The above parameters were chosen based on Supplementary Fig. S3.

### Dataset preparation and performance evaluation

To make a direct comparison between our algorithm and previous methods, we collected a group of well-established datasets with different levels of homology^5^,^11^,^12^,^13^,^14^,^18^. Cross-validation and independent testing were used to evaluate our various predictors. All of the parameters in our algorithms were determined based on the primary dataset (CSA223). The area under the receiver operating characteristic curve (AUC) was reported as a primary measure. We also calculated other metrics, including recall, precision, F1-score, accuracy (ACC), and Matthews correlation coefficient (MCC). Details can be found in Supplementary Methods.

## Results and Discussion

### Statistical analysis of novel structural features

**Figure 3:**
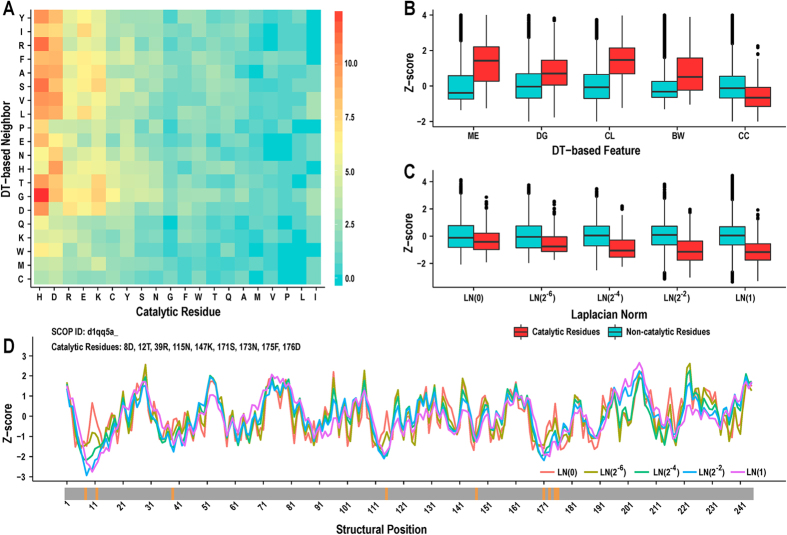
Statistical analysis of novel structural features. (**A**) Weight coefficients of residue pairs in the DT-based microenvironment of catalytic residues. Catalytic residues are sorted according to the percentages of different residue types. (**B**) A comparison of the distribution of DT-based features for catalytic and non-catalytic residues. ME: microenvironment score, DG: degree, CL: closeness, BW: betweenness, and CC: clustering coefficient. (**C**) A comparison of the distribution of LN-based geometric features for catalytic and non-catalytic residues. (**D**) Characterization of an enzyme structure (SCOP ID: d1qq5a_) by LNs at different scales. The positions marked in orange in the grey bar denote validated catalytic residues.

To obtain the DT-based microenvironment score (MEscore_DT_), we first computed an overall weight vector *W*_*DT*_ based on the CSA223 dataset^18^, which can be transformed into a 20 × 20 matrix. As shown in Fig. 3A, catalytic residues are enriched in charged and hydrophilic residues (H, D, R, E, K, C, and Y) but are depleted in hydrophobic residues. Our cluster analysis shows that neighboring residues have different preferences in the DT-based microenvironment. For instance, the neighbors of dominant catalytic residue types generally have greater weights and the neighborhoods of these residue types share more similar patterns. Han *et al*.^18^ recently utilized the distance-based criterion to generate the residue neighborhood. A high correlation exists between their weight vector and ours (Pearson’s correlation coefficient = 0.940). We further calculated the value of MEscore_DT_ for each residue in CSA223. Figure 3B shows that catalytic residues have significantly higher microenvironment scores than non-catalytic residues (*p-value* = 9.8E-123), implying that MEscore_DT_ can serve as an effective feature.

Previous studies commonly retrieved topological features using distance-based residue interaction networks. As shown in Fig. 3B, when using DT-based networks, catalytic residues possess clearly higher closeness values than other residues (*p-value* = 3.3E-144). A similar tendency holds for betweenness centrality (*p-value* = 4.7E-69), further suggesting the global importance of catalytic residues in the network. Additionally, catalytic residues tend to achieve greater values of degree (*p-value* = 3.0E-51) but smaller clustering coefficients (*p-value* = 3.8E-37). These results illustrate the fact that although catalytic residues physically interact with more residues, these neighbors are usually located in pocket or cleft regions and thus form few direct linkages among themselves due to the spatial gaps involved.

LN-based geometric features were also used for the first time to describe catalytic residues. As shown in Fig. 3C, the LNs of catalytic residues are consistently smaller than those of non-catalytic residues (*p-value* ≤ 8.3E-12) and the discrepancy is markedly increased as the scale factor becomes greater. This phenomenon indicates that catalytic residues prefer relatively concave locations in both the local and global structures of enzymes. However, when we directly used the five scale factors proposed by Li *et al*.^20^ to generate different LNs, the distribution comparison on the last four scales looks very similar (see Supplementary Fig. S2). In contrast, our scale factors, which were chosen based on systematic sampling, might result in more multifaceted features. Finally, we select an example from CSA223 and find that almost all of the catalytic residues appear at the valleys of LN curves (Fig. 3D), thus confirming the potential usefulness of LNs.

### Evaluation of feature predictors using the primary dataset

**Figure 4:**
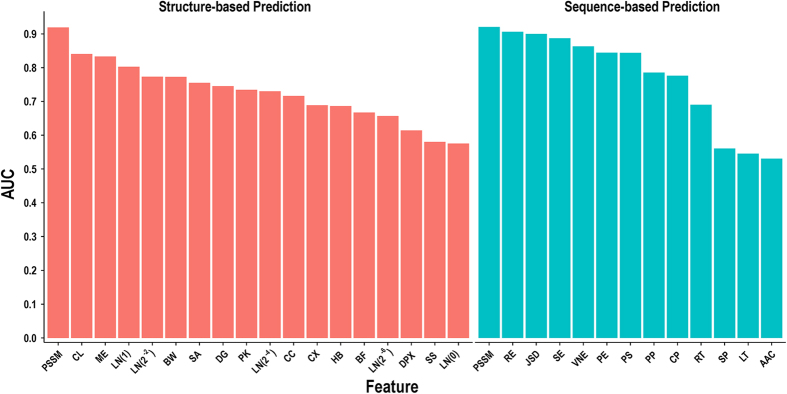
Performance of single attributes evaluated on CSA223. PSSM: position-specific scoring matrix, CL: closeness, ME: microenvironment score, LN: Laplacian norm, BW: betweenness, SA: solvent accessibility, DG: degree, PK: pocket, CC: clustering coefficient, CX: protrusion index, HB: hydrogen bonds, BF: B-factor, DPX: depth index, SS: secondary structure, RE: relative entropy, JSD: Jensen-Shannon divergence score, SE: Shannon entropy, VNE: von Neumann entropy, PE: property entropy, PS: predicted structural features, PP: physicochemical properties, CP: catalytic residue propensity, RT: residue type, SP: sequential position, LT: length, and AAC: amino acid composition.

In this section, we first built a group of SVM-based predictors using each single feature coupled with the structural or sequence microenvironment and evaluated these predictors using 5-fold cross-validation on the CSA223 dataset. Figure 4 shows that the PSSM feature achieves the best performance among the structure- and sequence-based predictors, yielding AUCs of 0.919 and 0.920, respectively. At the sequence level, the well-defined residue conservation scores provide competitive performances compared to PSSM. Although our novel descriptors are not as useful as evolutionary conservation features, they rank at the top of all structural features. For instance, the AUCs of DT-based closeness (CL), MEscore_DT_ (ME), and LN on the global scale (LN(1)) are 0.840, 0.833, and 0.802, respectively. The AUCs of the remaining structural and sequence descriptors generally range from 0.55 to 0.85, indicating that conventional features can detect catalytic signatures at different levels. In addition, we compared DT-based features with their distance-based counterparts. Supplementary Fig. S4 shows that the DT-based method achieves greater performance for degree and closeness measures but slightly weaker performance for the other measures. Overall, DT-based features can thus be considered as alternatives to distance-based features.

Next, we evaluated our final structural and sequence feature predictors using CSA223. As shown in Table 1, the performances of these two predictors are better than those obtained using the best single feature (PSSM-based) predictors. Both StrFeature and SeqFeature generate promising results, yielding AUCs of 0.952 and 0.949, respectively. In particular, without our novel descriptors, the structural feature predictor attains an AUC of 0.941 (see Supplementary Table S1). Obviously, both structure- and sequence-derived features can collaboratively contribute to catalytic residue prediction. We further combined these two predictors to assess whether they could complement each other. When we assign equal weights to the two predictors, the hybrid method SeqStrFeature achieves enhanced performance with an AUC of 0.960. This result indicates that the complementarity between structural and sequence information can be exploited to improve the prediction accuracy.

### Evaluation of template predictors using the primary dataset

**Figure 5:**
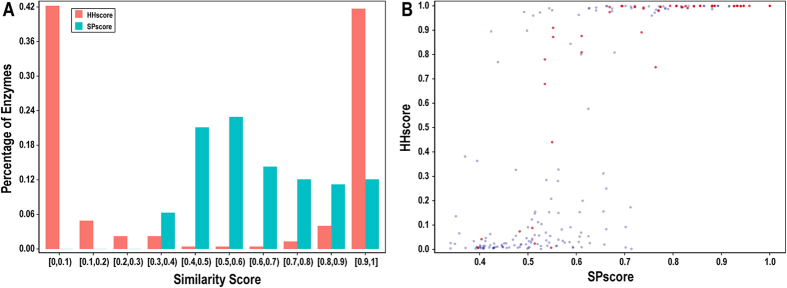
Similarity scores of the optimal templates for the primary dataset. (**A**) Distribution of SPscores and HHscores for CSA223. (**B**) Comparison of SPscores and HHscores for CSA223. The red dot suggests that our structural and sequence template predictors detect the same template for the query protein.

Most previous algorithms have used feature-based strategies to recognize catalytic residues. Herein, we developed a structural template predictor using SPalign and a sequence template predictor using HHblits, both of which were also tested on CSA223. The SPscore and HHscore distributions of the best templates are shown in Fig. 5A. For the structural method, 111 (49.7%) queries can achieve a reliable template with an SP-score of greater than 0.6, implying that the enzyme and its template probably share the same SCOP fold. In contrast, the sequence method exhibits a disjunctive distribution, in which 93 (41.7%) queries retrieve a good template, with HHscores of greater than 0.9. It is further shown in Fig. 5B that these two methods detect the same template for 64 (28.7%) proteins, most of which highly resemble the template in terms of both their structural and profile perspectives. After achieving the best template, the putative catalytic residues of each query can be annotated based on experimentally verified residues. As shown in Table 1, StrTemplate yields an F1-score of 0.277 and MCC of 0.274, whereas SeqTemplate achieves an F1-score of 0.292 and MCC of 0.314. These results confirm that both structural and sequence template predictors can recognize catalytic residues. More interestingly, the sequence predictor yields even better performance than the structural predictor, suggesting that sequence profile similarity is a powerful indicator to find remote templates of enzymes for which no structures are available.

### Evaluation of integrative predictors using the primary dataset

The rationale behind our method is to simultaneously use the complementary relationship between feature- and template-based strategies as well as that between structural and sequence information. As shown in Table 1, compared to their component predictors, our hybrid structural and sequence predictors (StrHunter and SeqHunter) achieve better performances, yielding AUCs of 0.958 and 0.954, respectively. Although SeqHunter performs slightly worse than StrHunter, the sequence-based hybrid strategy will have broader applications, because the number of known sequences is increasing faster than the solving of their structures. Our final predictor CRHunter represents the further integration of StrHunter and SeqHunter and yields an improved AUC of 0.967. The Venn diagrams presented in Supplementary Fig. S5 show that CRHunter fully utilizes different catalytic signatures given by the structure- and sequence-based modules and by their component predictors. In Supplementary Table S1, we confirm that the incorporation of our novel features can moderately improve the combined algorithms. In summary, it is reasonable to assume that CRHunter will serve as a useful catalytic residue detector in various scenarios.

### Evaluation of proposed predictors using the alternative datasets

**Figure 6:**
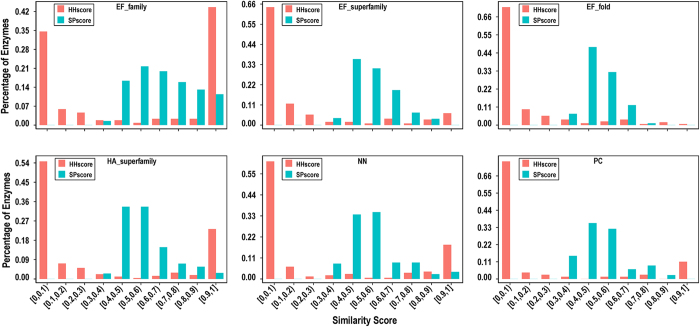
Distribution of the similarity scores of top-ranked templates for the alternative datasets. These six datasets have different levels of structural homology.

We checked our approach using the CSA223 dataset without redundancy at the sequence level. Furthermore, we evaluated our predictors by conducting 10-fold cross-validation on six datasets with different levels of structural homology. Focusing on the three datasets (EF series) collected by Youn *et al*.^13^, we can observe that our feature predictors achieve relatively stable performances and yield AUCs of greater than 0.915 (Table 2). As shown in Fig. 6, the number of enzymes that can retrieve an effective template decreases remarkably as the homological relationships between the entries in the dataset become weaker. Accordingly, the template methods yield poor results for the EF_superfamily and EF_fold datasets, which clearly indicates the weakness of template-based prediction when effective templates are lacking. As expected, StrHunter and SeqHunter do not provide better performance for these two datasets. In contrast, an obvious improvement is observed for the EF_family dataset due to the contribution from our template approaches. For the remaining three datasets, our template predictors retrieve reliable templates for a small group of all queries and correctly predict their catalytic residues, resulting in a slight improvement in the AUCs of StrHunter and SeqHunter compared to the feature-based methods. These results suggest that our algorithm has a strong adaptive capacity to the template quality of query proteins. More importantly, CRHunter continues to show optimal performance among all our predictors for different datasets and yields AUCs ranging from 0.926 to 0.952. Therefore, CRHunter is an intelligent prediction system that can automatically exploit the advantages of individual predictors.

### Evaluation of proposed predictors using the independent datasets

We further evaluated our algorithm with the external dataset T124^14^ and used the EF_fold dataset to train the prediction models. Table 3 shows that our sequence feature and template predictors generate similar performances with an MCC of approximately 0.270, whereas SeqHunter achieves a greater MCC of 0.303. Our structural module StrHunter also yields an improved MCC of 0.334 compared to its component predictors. Finally, CRHunter achieves the best performance with an MCC of 0.367 and AUC of 0.946, thus again confirming the usefulness and robustness of our integrative methodology. We then generated the predicted structures for the T124 dataset using I-TASSER^28^ with relaxed and strict template parameters. Table 3 shows that our three structural predictors remain robust for the predicted structures. The performances of these predictors are improved when we use more reliable structural models. For instance, the AUCs yielded by StrHunter are 0.923 and 0.933 for the low and high quality models, respectively. Compared to sequence-based methods, structural model-based prediction does not show significant advantages. However, when we merge these two types of information, CRHunter exhibits enhanced performance, yielding AUCs of 0.940 and 0.942 for these two datasets. This fusion makes our algorithm more powerful in the case of queries comprising only sequence information. Moreover, we replaced the EF_fold dataset with the CSA223 dataset and obtained similar results, as shown in Supplementary Table S2.

### Comparison with other prediction algorithms

We systematically evaluated our method using a group of widely used datasets. In this way, we can compare our performance with the results reported from previous literature. Based on CSA223, Han *et al*.^18^ integrated the distance-based microenvironment score, distance to the centroid, and residue conservation to predict catalytic residues, yielding an AUC of 0.920. The FEATURE algorithm^29^ attained an AUC of 0.874 on the same dataset. Clearly, CRHunter generates superior performance, because it yields an AUC of 0.967. Using our alternative and external datasets, we further compared CRHunter with other algorithms. In Table 4, when the precisions of CRHunter are equal to those of the competing methods, our recalls are generally higher than the recalls of the competing methods and CRpred^14^. If the recalls are fixed, the precisions of CRHunter are also better. Similarly, we considered the performance of CRpred as the baseline and compared the results of CatANalyst^30^ and CRHunter in Supplementary Table S3. Compared to the structural feature-based method CatANalyst, our integrative algorithm yields greater measures when using the EF_family, HA_superfamily, and T124 datasets. The incorporation of template predictors probably makes an important contribution to our superior performance. For the other three datasets with lower homology, these two algorithms achieve comparable performance. These tables also show that SeqHunter achieves competitive or better results at the sequence level than CRpred does. In summary, our algorithm exhibits superiority over the state-of-the-art algorithms due to its combination of multifaceted information.

### Application to structural genomics targets

**Figure 7:**
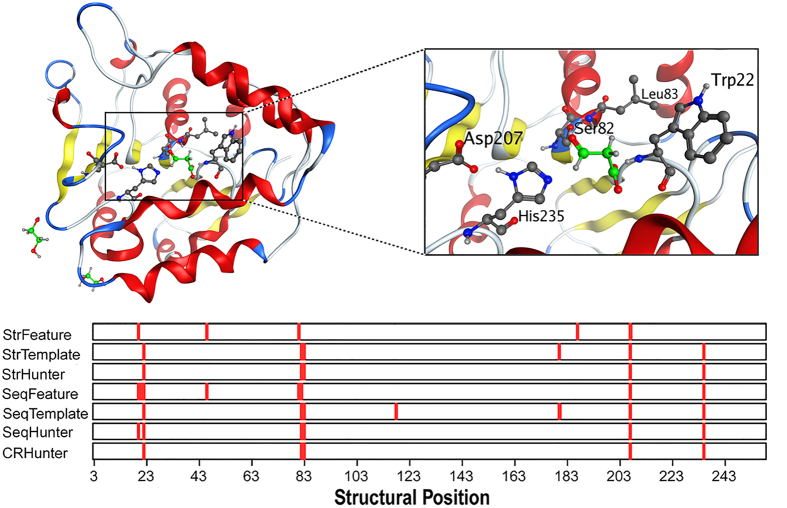
Prediction results obtained for the BioH protein (PDB ID: 1M33_A) by our different predictors. The positions in red in the bars denote the predicted catalytic residues.

Finally, we applied our approach to the SG2332 dataset comprising 2332 protein structures with unknown functions. Note that all of the predictors were trained on CSA223, and only three entries shared more than 30% sequence identity with CSA223. As shown in Supplementary Fig. S6, we achieve at least one positive prediction in 1704 protein structures, which include 6746 putative catalytic residues. We also notice that the distribution of putative catalytic residues in SG2332 strongly correlates with the distribution of validated catalytic residues in CSA223 (Pearson’s correlation coefficient = 0.958), indicating that our predictions are generally reliable. To further show the power of our method, we selected the BioH protein (PDB ID: 1M33_A) as a representative structure. The original reference annotated a putative catalytic triad (Ser82, His235, and Asp207) in BioH by aligning this protein against active site templates with TESS and experimentally validated that Ser82 probably plays an important role in the enzymatic activity^31^. As revealed in Fig. 7, our component predictors all output several positive predictions in BioH, which generally cover the potential catalytic triad. Both StrTemplate and SeqTemplate retrieve the same template for BioH (SCOP ID: d1ehya_), which has high structural but low sequence similarity with this query (SPscore = 0.97 and sequence identity = 21%). Through merging the outputs of different predictors, CRHunter eliminates the potential false positives and returns five possible catalytic residues (Trp22, Ser82, Leu83, Asp207, and His235). The possible catalytic functions of Trp22 and Leu83 are especially worthy of further study. The precompiled results for SG2332 are provided in the dataset page of our server.

### CRHunter server

CRHunter is freely available at http://www.bioinfo-hzau.cc/CRHunter/ and was developed in PHP, Perl-CGI, JpGraph, and Jmoe. We provide different prediction methods based on three types of information (sequence, structure, and structural model) to identify putative catalytic residues in query proteins. An example of the results obtained from our server is presented in Supplementary Fig. S7.

## Conclusions

To our knowledge, this is the first time that an integrative algorithm simultaneously uses structure- and sequence-based feature and template strategies to predict catalytic residues. First, several novel descriptors based on the Delaunay triangulation and Laplacian transformation of enzyme structures were used in our structural feature predictor. Compared with traditional structural attributes, these new features not only yield better performance but also provide orthogonal information. Alternatively, our sequence feature predictor achieves competitive results compared to its structural equivalent, thus demonstrating its predictive power and possible broader applications. Regarding template-based prediction, both our structural and sequence predictors can find effective templates for approximately 45% of all enzymes without sequence redundancy and can therefore be used to transfer experimentally verified catalytic residues. By using an effective combination of these methods, our hybrid algorithm CRHunter outperforms the structural and sequence prediction modules as well as their component predictors. When they are applied to datasets with different levels of structural homology, our feature predictors generate relatively stable performance, whereas our template predictors yield poor results as the homological relationships become weak. CRHunter, however, continues to achieve the best performance among all our proposed predictors for these datasets. Through independent testing, we find that our predictors are robust and that the integrative strategy can be applied to structural model-based catalytic residue prediction as well as its sequence- and structure-based counterparts. Compared to other prediction methods, CRHunter is generally superior for various datasets. Finally, the application of this method to structural genomics targets will provide valuable insights into solved structures with unknown functions.

## Additional Information

**How to cite this article**: Sun, J. *et al*. CRHunter: integrating multifaceted information to predict catalytic residues in enzymes. *Sci. Rep.* **6**, 34044; doi: 10.1038/srep34044 (2016).

## Supplementary Material

Supplementary Information

## Figures and Tables

**Table 1:** Performance of proposed predictors on primary dataset.

Method^a^	Recall	Precision	F1	ACC	MCC	AUC
StrFeature	0.553 (0.036)^b^	0.200 (0.011)	0.292 (0.013)	0.972 (0.001)	0.320 (0.015)	0.952 (0.004)
StrTemplate	0.239 (0.028)	0.329 (0.028)	0.277 (0.029)	0.987 (0.001)	0.274 (0.029)	N/A
StrHunter	0.568 (0.021)	0.247 (0.017)	0.342 (0.017)	0.977 (0.001)	0.364 (0.015)	0.958 (0.004)
SeqFeature	0.545 (0.021)	0.201 (0.010)	0.293 (0.013)	0.973 (0.001)	0.320 (0.014)	0.949 (0.003)
SeqTemplate	0.212 (0.030)	0.485 (0.038)	0.292 (0.035)	0.990 (0.001)	0.314 (0.033)	N/A
SeqHunter	0.551 (0.021)	0.221 (0.012)	0.316 (0.015)	0.975 (0.001)	0.339 (0.016)	0.954 (0.004)
SeqStrFeature	0.577 (0.023)	0.251 (0.015)	0.349 (0.015)	0.978 (0.001)	0.370 (0.014)	0.960 (0.002)
CRHunter	0.579 (0.028)	0.302 (0.020)	0.396 (0.021)	0.982 (0.001)	0.409 (0.021)	0.967 (0.002)

^a^StrFeature and SeqFeature represent our feature predictors based on structural and sequence information, respectively. StrTemplate and SeqTemplate represent our template predictors based on structure and profile alignments, respectively. StrHunter (SeqHunter) is the combination of StrFeature (SeqFeature) and StrTemplate (SeqTemplate). SeqStrFeature is the fusion of SeqFeature and StrFeature. CRHunter is our final prediction algorithm.

^b^Standard errors of various measures are shown in parentheses.

**Table 2:** Performance of proposed predictors on alternative datasets.

Dataset	Method^a^	Recall	Precision	F1	ACC	MCC	AUC
EF_family	Feature (Str|Seq)	0.503^b^ (0.486)^c^	0.208 (0.206)	0.292 (0.289)	0.973 (0.974)	0.311 (0.305)	0.937 (0.931)
	Template(Str|Seq)	0.229 (0.200)	0.303 (0.451)	0.260 (0.276)	0.986 (0.989)	0.256 (0.295)	N/A
	Hunter(Str|Seq)	0.489 (0.509)	0.258 (0.226)	0.336 (0.313)	0.979 (0.976)	0.345 (0.328)	0.941 (0.936)
	CRHunter	0.497	0.305	0.376	0.982	0.380	0.949
EF_superfamily	Feature(Str|Seq)	0.509 (0.452)	0.211 (0.193)	0.297 (0.270)	0.973 (0.972)	0.315 (0.283)	0.938 (0.924)
	Template(Str|Seq)	0.032 (0.023)	0.056 (0.149)	0.040 (0.040)	0.983 (0.988)	0.034 (0.054)	N/A
	Hunter(Str|Seq)	0.506 (0.536)	0.189 (0.163)	0.274 (0.250)	0.970 (0.964)	0.296 (0.282)	0.937 (0.925)
	CRHunter	0.523	0.218	0.307	0.974	0.326	0.944
EF_fold	Feature(Str|Seq)	0.448 (0.363)	0.210 (0.182)	0.282 (0.241)	0.971 (0.971)	0.292 (0.243)	0.918 (0.907)
	Template(Str|Seq)	0.017 (0.018)	0.030 (0.124)	0.021 (0.031)	0.981 (0.986)	0.013 (0.042)	N/A
	Hunter(Str|Seq)	0.505 (0.497)	0.178 (0.160)	0.259 (0.241)	0.964 (0.961)	0.283 (0.265)	0.918 (0.907)
	CRHunter	0.504	0.211	0.293	0.970	0.311	0.926
HA_superfamily	Feature(Str|Seq)	0.539 (0.497)	0.198 (0.182)	0.289 (0.266)	0.974 (0.973)	0.316 (0.290)	0.944 (0.933)
	Template(Str|Seq)	0.103 (0.091)	0.150 (0.326)	0.121 (0.141)	0.986 (0.990)	0.117 (0.168)	N/A
	Hunter(Str|Seq)	0.557 (0.556)	0.186 (0.174)	0.278 (0.265)	0.972 (0.970)	0.310 (0.299)	0.944 (0.936)
	CRHunter	0.575	0.233	0.330	0.977	0.356	0.952
NN	Feature(Str|Seq)	0.475 (0.468)	0.207 (0.201)	0.286 (0.280)	0.974 (0.974)	0.301 (0.295)	0.935 (0.932)
	Template(Str|Seq)	0.108 (0.088)	0.141 (0.393)	0.122 (0.139)	0.983 (0.989)	0.115 (0.177)	N/A
	Hunter(Str|Seq)	0.541 (0.543)	0.196 (0.178)	0.285 (0.267)	0.971 (0.968)	0.312 (0.298)	0.939 (0.934)
	CRHunter	0.551	0.243	0.335	0.977	0.354	0.949
PC	Feature(Str|Seq)	0.478 (0.383)	0.214 (0.181)	0.285 (0.242)	0.972 (0.972)	0.302 (0.249)	0.936 (0.923)
	Template(Str|Seq)	0.061 (0.019)	0.088 (0.075)	0.072 (0.030)	0.982 (0.988)	0.064 (0.034)	N/A
	Hunter(Str|Seq)	0.524 (0.469)	0.189 (0.161)	0.274 (0.234)	0.967 (0.964)	0.299 (0.257)	0.937 (0.924)
	CRHunter	0.493	0.231	0.306	0.974	0.321	0.945

^a^Feature(Str|Seq) denotes our feature predictor based on structural or sequence information. Template(Str|Seq) denotes our template predictor based on structure or profile alignment. Hunter(Str|Seq) is the combined structural or sequence module. CRHunter is our final prediction algorithm.

^b^Results generated by structure-based predictors.

^c^Results generated by sequence-based predictors.

**Table 3:** Performance of proposed predictors on independent datasets.

Data type^a^	Method^b^	Recall	Precision	F1	ACC	MCC	AUC
Native sequence	SeqFeature	0.470 (0.035)^c^	0.174 (0.014)	0.254 (0.018)	0.978 (0.001)	0.277 (0.020)	0.929 (0.008)
	SeqTemplate	0.172 (0.023)	0.416 (0.037)	0.243 (0.028)	0.992 (0.000)	0.263 (0.028)	N/A
	SeqHunter	0.475 (0.037)	0.204 (0.019)	0.286 (0.021)	0.981 (0.002)	0.303 (0.021)	0.930 (0.009)
Native structure	StrFeature	0.496 (0.030)	0.189 (0.014)	0.274 (0.017)	0.979 (0.001)	0.297 (0.018)	0.943 (0.007)
	StrTemplate	0.206 (0.024)	0.262 (0.029)	0.230 (0.025)	0.989 (0.001)	0.227 (0.026)	N/A
	StrHunter	0.501 (0.035)	0.233 (0.021)	0.318 (0.024)	0.983 (0.001)	0.334 (0.024)	0.945 (0.008)
	CRHunter	0.488 (0.037)	0.286 (0.023)	0.361 (0.024)	0.986 (0.001)	0.367 (0.024)	0.946 (0.008)
High quality model	StrFeature	0.449 (0.032)	0.176 (0.015)	0.253 (0.018)	0.979 (0.001)	0.272 (0.018)	0.931 (0.007)
	StrTemplate	0.187 (0.022)	0.228 (0.026)	0.206 (0.023)	0.988 (0.001)	0.201 (0.023)	N/A
	StrHunter	0.443 (0.033)	0.209 (0.017)	0.284 (0.020)	0.982 (0.001)	0.296 (0.020)	0.933 (0.008)
	CRHunter	0.454 (0.037)	0.275 (0.022)	0.343 (0.022)	0.986 (0.001)	0.347 (0.022)	0.942 (0.008)
Low quality model	StrFeature	0.385 (0.033)	0.160 (0.014)	0.226 (0.018)	0.979 (0.001)	0.239 (0.019)	0.919 (0.008)
	StrTemplate	0.185 (0.021)	0.248 (0.027)	0.212 (0.022)	0.989 (0.000)	0.209 (0.023)	N/A
	StrHunter	0.396 (0.032)	0.202 (0.019)	0.268 (0.021)	0.983 (0.001)	0.275 (0.021)	0.923 (0.009)
	CRHunter	0.406 (0.035)	0.267 (0.023)	0.322 (0.023)	0.986 (0.001)	0.323 (0.023)	0.940 (0.008)

^a^High and low quality models denote the structures modelled by I-TASSER with relaxed and strict sequence identity cutoffs (90% and 30%), respectively.

^b^Annotations of different methods are the same as those in Table 1.

^c^Standard errors of various measures are shown in parentheses.

**Table 4:** Comparison with other prediction methods.

Method^a^	Reported measure^b^	EF family	EF superfamily	EF fold	HA superfamily	NN	PC
Competing method	Recall (Precision)	0.570 (0.185)^c^	0.539 (0.169)^c^	0.511 (0.171)^c^	0.293 (0.165)^d^	0.560 (0.140)^e^	0.900 (0.070)^f^
CRpred	Recall (Precision)	0.583 (0.195)^g^	0.521 (0.159)^g^	0.480 (0.161)^g^	0.497 (0.247)^g^	0.659 (0.180)^g^	0.845 (0.056)^g^
SeqHunter	Recall (Precision)	0.614 (0.209)	0.520 (0.161)	0.409 (0.156)	0.576 (0.274)	0.662 (0.174)	0.838 (0.047)
CRHunter	Recall (Precision)	0.712 (0.273)	0.687 (0.215)	0.627 (0.210)	0.696 (0.332)	0.764 (0.240)	0.921 (0.076)

^a^SeqHunter is the combination of our sequence-based feature and template methods. CRHunter is our final prediction algorithm.

^b^Recall (precision) values of CRpred, SeqHunter, and CRHunter are reported when their precision (recall) values are equal to those of the competing methods.

^c^Results on the EF_family, EF_superfamily, and EF_fold datasets reported from Youn *et al*.^13^.

^d^Results on the HA_superfamily dataset reported from Chea *et al*.^5^.

^e^Results on the NN dataset reported from Gutteridge *et al*.^11^.

^f^Results on the PC dataset reported from Petrova and Wu.^12^.

^g^Results on the above six datasets reported from Zhang *et al*.^14^.
