# Supplementary material for: CRHunter: integrating multifaceted information to predict catalytic residues in enzymes
Source: Sci Rep. 2016 Sep 26;6:34044. doi: 10.1038/srep34044 (PMC5036049; doi:10.1038/srep34044)
Supplement: Supplementary Information [file srep34044-s1.pdf]

Supplementary Information

CRHunter: integrating multifaceted information to predict catalytic residues in enzymes

Jun Sun, Jia Wang, Dan Xiong, Jian Hu, Rong Liu*

Hubei Key Laboratory of Agricultural Bioinformatics, College of Informatics, Huazhong Agricultural University, Wuhan 430070, P. R. China.

* Corresponding author

Email: liurong116@mail.hzau.edu.cn

Tel: +86-27-87280877

Fax: +86-27-87280877

Supplementary Methods

Structure-based feature generation

In addition to the novel features derived from the Delaunay triangulation and Laplacian characterization of protein structures, conventional structure-based attributes, including solvent accessibility, depth and protrusion indices, pocket information, secondary structure, hydrogen bonds, and B-factor, were also used in the current study.

Residue solvent accessibility

Accessible surface area is the exposed region of a molecule that is accessible to solvents. The NACCESS program[1] was utilized to calculate the solvent accessibility of each residue, which includes the absolute and relative accessible surface areas of total atoms, total side-chain atoms, nonpolar side-chain atoms, polar side-chain atoms, and total main-chain atoms.

Depth and protrusion indices

Depth and protrusion indices are geometric features that describe the local concavity and convexity of a given protein structure, respectively. We used the PSAIA software[2] with default parameters to generate the average depth and protrusion indices of all atoms in each residue.

Pocket information

Catalytic residues generally locate in the pocket or cleft regions in enzyme structures. We thus detected all pockets in the query using the Fpocket2 program[3]. The location of each residue was then divided into four categories, including the largest pocket, the second or third largest pocket, the fourth to ninth largest pocket, and none of the above pockets.

Secondary structure

Secondary structure can be utilized to reflect the local conformation of protein structures. The secondary structure of each residue was assigned by the DSSP program[4]. Residues in H (α-helix), G (3/10-helix), and I (π-helix) are considered to be in helical conformation, and those in E (β-strand) and B (β-bridge) are in sheet conformation. All remaining residues are in coil conformation.

Hydrogen bonds

Hydrogen bonds play important roles in maintaining the conformation of catalytic residues serving as donors or acceptors. We calculated hydrogen bonds using HBPLUS[5] and achieved three features including the number of hydrogen bonds between side-chain atoms in query residue and other atoms in a protein, the number of hydrogen bonds between main-chain atoms in query residue and other atoms, and the number of hydrogen bonds including any atom in query residue.

B-factor

B-factor, also called temperature factor, is a measure of atomic thermal motion and disorder. In this work, we used the B-factor of alpha carbon to represent the flexibility of each residue.

Sequence-based feature generation

As the complements of structure-based features, we extracted a variety of sequence-based descriptors to characterize each residue, such as residue type, position-specific scoring matrix, residue conservation scores, predicted structural features, physicochemical properties, catalytic residue propensity, sequential position, and global sequence features.

Residue type

The identity of each residue can be used to check its catalytic signature. Each sequence position was thus encoded by a vector composed of 20 elements where the target residue type was set to 1 and the remainder was set to 0.

Position-specific scoring matrix (PSSM)

PSSM is a sequence profile comprising the evolutionary information of a protein sequence. For each query, we performed a search against NR database from NCBI using the PSI-BLAST program[6] with the parameters $j = 3$ and $e = 0.001$ to generate this profile.

Residue conservation scores

In addition to PSSM, the conservation scores of each residue can also be used to measure its evolutionary signatures. Here we implemented five entropy-based scores proposed by Capra and Singh[7], such as Shannon entropy[8], property entropy[9,10], von Neumann entropy[11], relative entropy[12], and Jensen-Shannon divergence[7]. These features were generated based on the weighted observed percentages matrix output by PSI-BLAST.

Predicted structural features

Predicted structural features can offer useful information in the absence of native structures. We generated the predicted secondary structure, solvent accessible surface area, and backbone dihedral angles by SPINEX[13] and extracted the predicted disorder score by SPINED[14].

Physicochemical properties

The physicochemical attributes of each residue can affect its catalytic performance. In this work, we retrieved nine amino acid indices from the AAindex database[15], including number of atoms, number of electrostatic charge, number of potential hydrogen bonds, hydrophobicity, hydrophilicity, isoelectric point, mass, expected number of contacts within 14Å sphere, and electron-ion interaction potential, to reflect the specific nature of each residue type.

Catalytic residue propensity

It is well known that residues have different tendency to be involved in catalytic activity. We thus calculated the catalytic residue propensity of each residue type which is defined as the ratio between the amino acid frequency in catalytic residues and that in the whole sequence.

Sequential position

Sequential position can reflect the relative location of each residue in a given sequence. To this end, we extracted two features proposed by Chen et al.[16], including terminus indicator and secondary structure segment indicators. The former is used to check whether a residue locates

in the N- or C-terminus, whereas the latter checks whether a predicted helix or sheet segment exists in the neighborhood of a given residue. More details can be found in the original reference.

Global sequence features

The global information of a given sequence was captured by two features, including the sequence length and the amino acid composition of each sequence.

Dataset preparation

Primary dataset

We used the dataset collected by Han et al.[17], composed of 223 enzymes (CSA223), as the primary dataset to construct and evaluate our method. All entries in CSA223 were extracted from the SCOP database[18] and their structures cover the four major structural classes (i.e., all-α, all-β, α+β, α/β). The sequence identity of any chain pair is less than 30%. Catalytic residues were collected from scientific literatures in the Catalytic Site Atlas. We finally attained 630 catalytic residues in this non-redundant dataset and the remaining 60658 residues were considered as non-catalytic residues.

Alternative datasets

Besides the CSA223 dataset, we further checked the robustness of our method using other well-established datasets, including EF_family, EF_superfamily, and EF_fold from Youn et al.[19], HA_superfamily from Chea et al.[20], NN from Gutteridge et al.[21], and PC from Petrova and Wu[22]. These datatsets were non-redundant at different structural levels. For instance, the entries of EF series were from the representative families, superfamilies, and folds in terms of the SCOP classification.

Independent test datasets

The T124 dataset curated by Zhang et al.[23] was applied to independent testing in our work. All these 124 entries came from the HA_superfamily dataset and shared less than 30% sequence identity with any chain from the EF_fold dataset serving as the training set. In addition to the native structures of these enzymes, we also attempted to estimate whether our algorithm can be applicable to the predicted structures. To this end, we prepared another two datasets T124_M90 and T124_M30, in which the structures were modelled by the I-TASSER software[24] with relaxed and stringent sequence identity cutoffs (90% and 30%), respectively. Structural model having the best C-score was chosen as a representative for each entry. The mean RMSD between the native structures and high quality models (T124_M90) is 1.86Å, while the value between the native structures and low quality models (T124_M30) is 2.48Å, suggesting that the quality of predicted structures is generally accepted.

Structural genomics dataset

We also built a structural genomics targets dataset called SG2332 which was selected from the Oct 2015 PDB release. We first searched the classification keyword ‘structural genomics’ in the PDB database and attained 2577 PDB entries having structural information but without functional annotations. The retrieved entries were then split into 5505 individual chains, which were further clustered at a 90% sequence identity cutoff using the CD-HIT program[25]. We

randomly selected one representative from each cluster and finally achieved 2332 chains.

Performance evaluation

To make a direct comparison with existing methods, we evaluated our predictors using 5-fold cross-validation on the primary dataset (CSA223) and 10-fold cross-validation on the alternative datasets (e.g., EF_family, EF_superfamily, etc.), respectively. For conducting *n*-fold cross-validation, the target dataset was first separated into *n* subsets with an equal number of chains. Then one subset was used as the test set, while the rest was used as the training set. This procedure was repeated *n* times, in which each subset was tested in turn, to estimate the average performance. All parameters in our algorithm were determined using 5-fold cross-validation on CSA223. Moreover, the native and predicted structures in T124 were considered as the external data to assess our predictors trained on the EF_fold and CSA223 datasets. As a primary measure of prediction performance, the area under the receiver operating characteristic curve (AUC) was calculated. We also computed other well-established metrics including recall, precision, F1-score, accuracy (ACC), and Matthews correlation coefficient (MCC) as below. TP, TN, FP, and FN denote the numbers of true positives, true negatives, false positives, and false negatives, respectively.

$$\text{Recall} = \frac{\text{TP}}{\text{TP} + \text{FN}}$$

$$\text{Precision} = \frac{\text{TP}}{\text{TP} + \text{FP}}$$

$$\text{F1-score} = \frac{2 \times \text{Recall} \times \text{Precision}}{\text{Recall} + \text{Precision}}$$

$$\text{Accuracy} = \frac{\text{TP} + \text{TN}}{\text{TP} + \text{FN} + \text{TN} + \text{FP}}$$

$$\text{MCC} = \frac{\text{TP} \times \text{TN} - \text{FP} \times \text{FN}}{\sqrt{(\text{TP} + \text{FN})(\text{TP} + \text{FP})(\text{TN} + \text{FP})(\text{TN} + \text{FN})}}$$

Additionally, we provided the standard error (SE) for all measures used in this work. For the 5-fold cross-validation (CSA223), the SE was estimated based on the performance of the five subsets. For the independent testing (T124), the bootstrapping algorithm was applied to the estimation of SE. We randomly selected (with replacement) a set of 124 chains from the T124 dataset and repeated this procedure 100 times. The SE was then estimated using the results of these bootstrap datasets.

Supplementary Figures and Tables

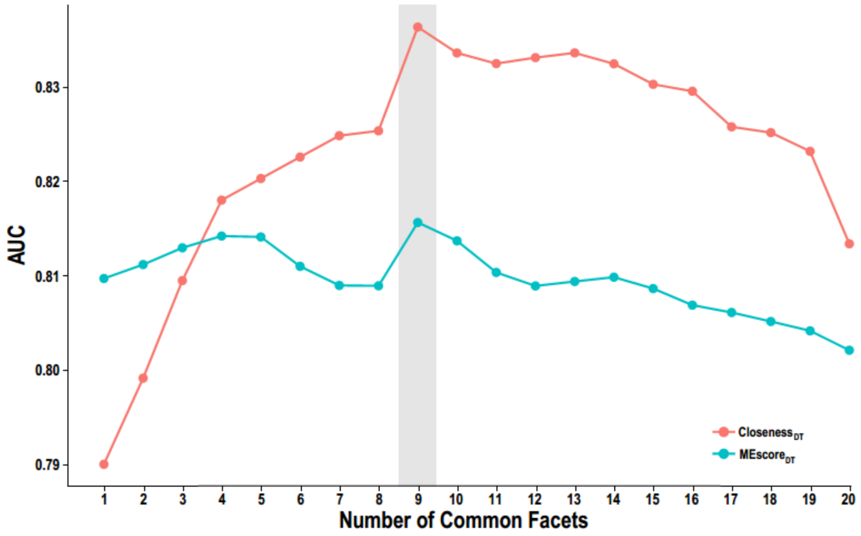

Figure S1. Selection of the optimal parameter for residue microenvironment. We utilized the number of shared facets in residue pairs as a constraint to generate different microenvironments for each residue. When the number is no less than 9, both DT-based MEscore and closeness yield optimal performance. Note that the catalytic function of a query residue was identified if its attribute value is larger than a given cutoff.

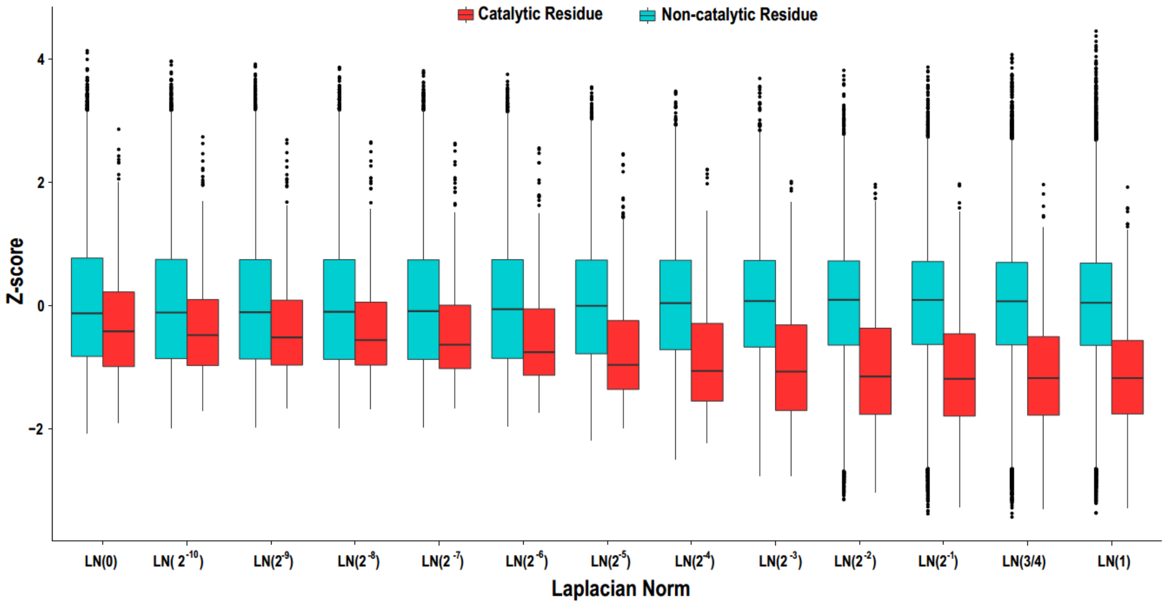

Figure S2. A comparison of the distribution of Laplacian norms by systematical sampling of different scale factors. The distribution on the last four scales (2^{-2}, 2^{-1}, 3/4, 1) proposed by Li et al.[26] looks very similar. We thus selected five representative scalar factors with an increased distribution discrepancy, which might contribute to more multifaceted LN features.

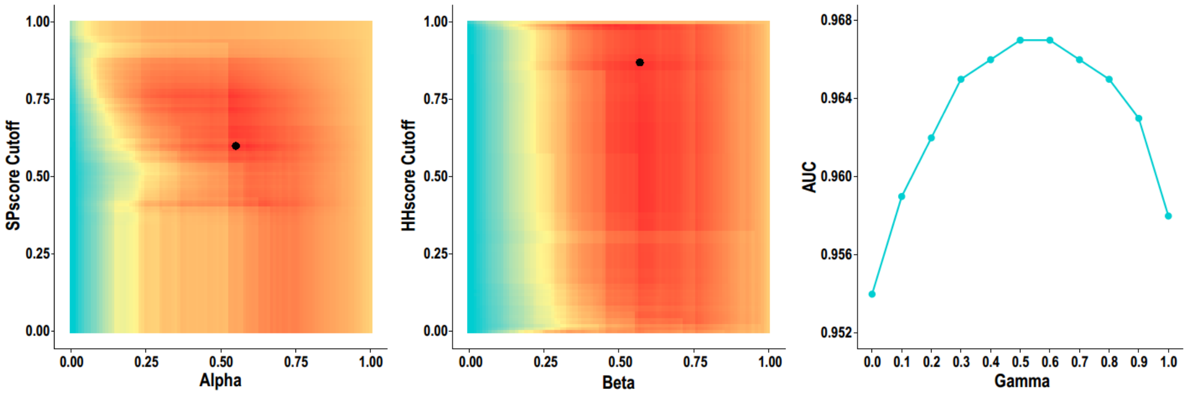

Figure S3. Selection of the optimal parameters for our hybrid algorithm. In the heat maps, the color is changed according to the AUC value of our structural or sequence prediction module with different parameter combinations (orange/yellow/cyan = high/medium/low), and the black dot denotes the highest AUC value.

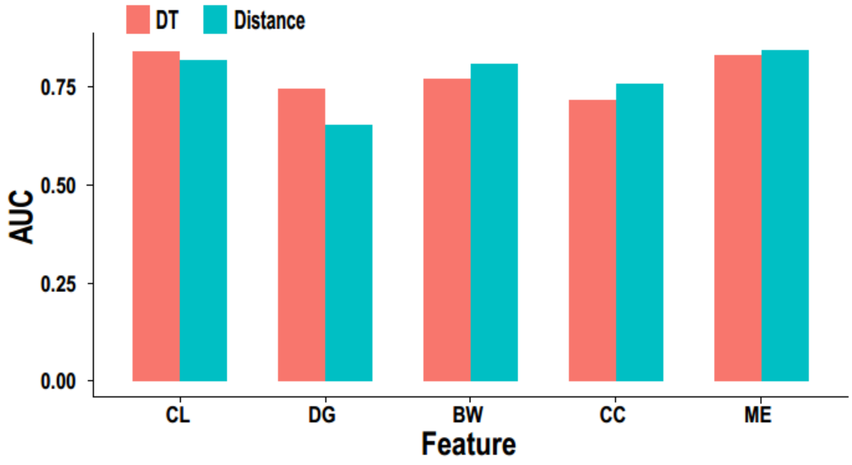

Figure S4. Performance of DT- and distance-based microenvironment score and topological features. Distance-based method was implemented based on Han et al.[17]. CL: closeness, DG: degree, BW: betweenness, CC: clustering coefficient, and ME: microenvironment score.

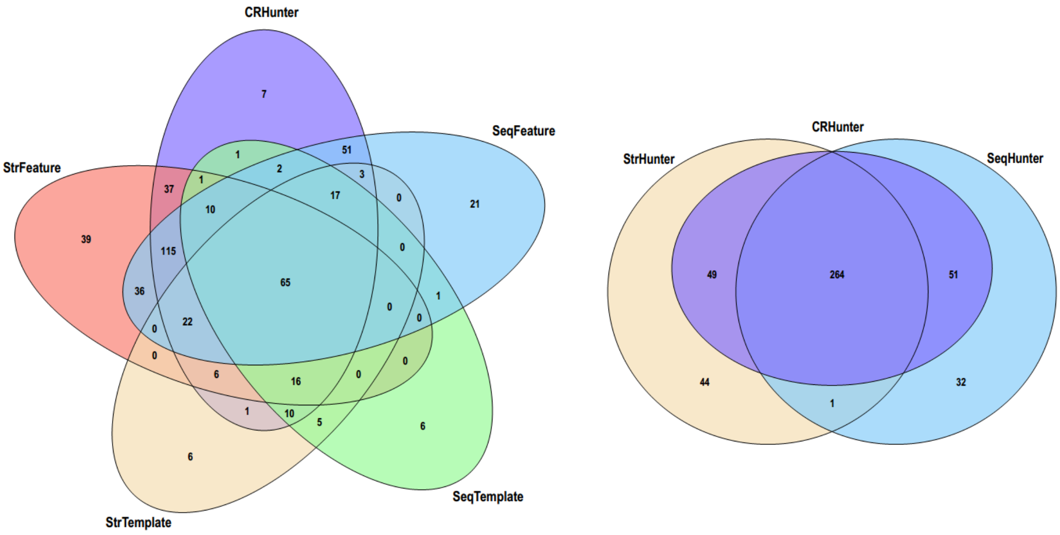

Figure S5. Venn diagram of true positives output by our proposed predictors. The total numbers of true positives (positives) of StrFeature, SeqFeature, StrTemplate, SeqTemplate, StrHunter, SeqHunter, and CRHunter are 347 (1768), 343 (1713), 151 (452), 134 (273), 358 (1487), 348 (1575), and 364 (1220), respectively. Obviously, CRHunter has considerable overlap with other predictors, suggesting that our hybrid algorithm effectively utilizes various catalytic signatures output by our structural and sequence prediction modules and by their component predictors.

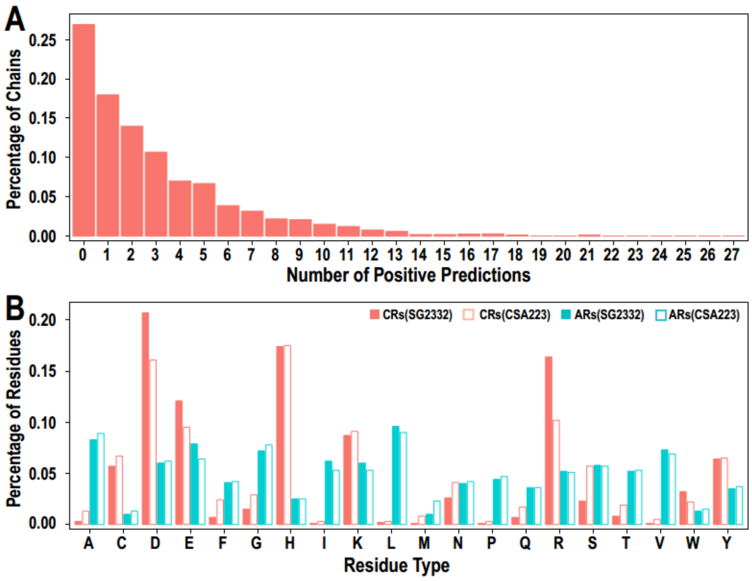

Figure S6. Prediction results for structural genomics targets (SG2332). (A) Distribution of predicted catalytic residues by chains. (B) Amino acid distribution of catalytic and all residues for the CSA223 and SG2332 datasets. CRs denotes catalytic residues, and ARs denotes all residues.

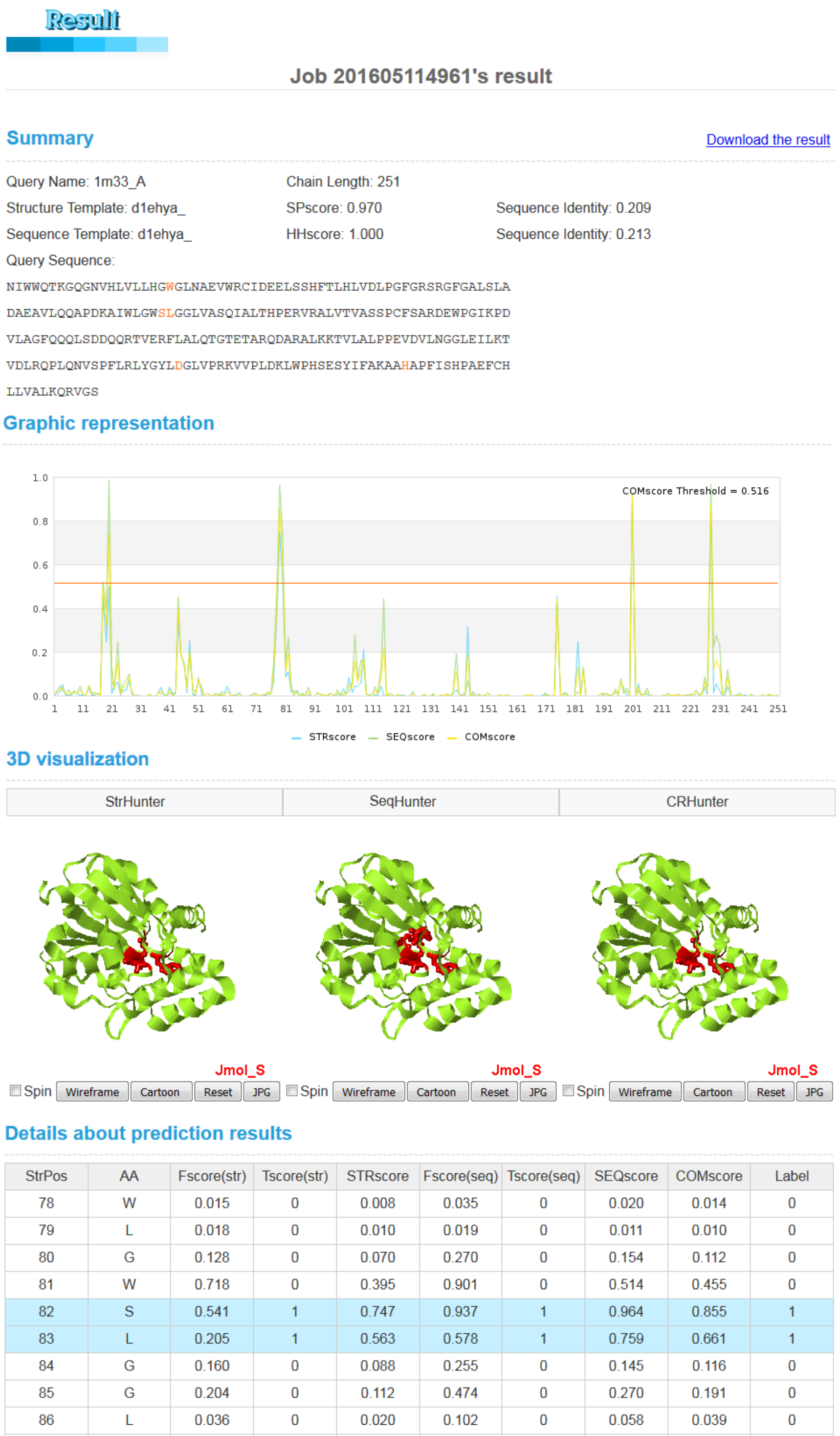

StrPos	AA	Fscore(str)	Tscore(str)	STRscore	Fscore(seq)	Tscore(seq)	SEQscore	COMscore	Label
78	W	0.015	0	0.008	0.035	0	0.020	0.014	0
79	L	0.018	0	0.010	0.019	0	0.011	0.010	0
80	G	0.128	0	0.070	0.270	0	0.154	0.112	0
81	W	0.718	0	0.395	0.901	0	0.514	0.455	0
82	S	0.541	1	0.747	0.937	1	0.964	0.855	1
83	L	0.205	1	0.563	0.578	1	0.759	0.661	1
84	G	0.160	0	0.088	0.255	0	0.145	0.116	0
85	G	0.204	0	0.112	0.474	0	0.270	0.191	0
86	L	0.036	0	0.020	0.102	0	0.058	0.039	0

Figure S7. A snapshot of prediction results from our server. As shown in this figure, the results will be displayed from four perspectives. The first section provides summary information about the query protein and its optimal template. The second section shows graphical representation of prediction results, in which STRscore, SEQscore, and COMscore denote the prediction scores output by our structure-based module, sequence-based module, and integrative algorithm, respectively. The third section provides three-dimensional visualization of prediction results, in which the putative catalytic residues are highlighted in red sticks. The last section includes the details about prediction results, such as the outputs from our different predictors.

Table S1. Performance of proposed predictors without novel features

Methods[a]	Recall	Precision	F1	ACC	MCC	AUC
StrFeature	0.553	0.200	0.292	0.972	0.320	0.952
StrFeature-ML	0.544	0.200	0.290	0.972	0.317	0.949
StrFeature-MTL	0.489	0.181	0.263	0.972	0.285	0.941
SeqStrFeature	0.577	0.251	0.349	0.978	0.370	0.960
SeqStrFeature-ML	0.566	0.250	0.346	0.978	0.366	0.959
SeqStrFeature-MTL	0.533	0.241	0.331	0.978	0.348	0.956
StrHunter	0.568	0.247	0.342	0.977	0.364	0.958
StrHunter-ML	0.549	0.236	0.328	0.977	0.349	0.956
StrHunter-MTL	0.538	0.237	0.327	0.977	0.346	0.950
CRHunter	0.579	0.302	0.396	0.982	0.409	0.967
CRHunter-ML	0.569	0.297	0.390	0.982	0.402	0.966
CRHunter-MTL	0.568	0.295	0.388	0.982	0.401	0.963

[a] Annotations of different methods are the same as those in Table 1. Predictor-ML denotes the predictor without the Microenvironment score and Laplacian norms. Predictor-MTL denotes the predictor without the Microenvironment score, Topological features, and Laplacian norms.

Table S2. Performance of proposed predictors on independent datasets (trained on CSA223)

Data type[a]	Method[b]	Recall	Precision	F1	ACC	MCC	AUC
Native sequence	SeqFeature	0.422	0.165	0.237	0.978	0.254	0.926
	SeqTemplate	0.156	0.411	0.226	0.992	0.250	N/A
	SeqHunter	0.430	0.169	0.242	0.979	0.260	0.927
Native structure	StrFeature	0.528	0.182	0.271	0.977	0.301	0.943
	StrTemplate	0.161	0.257	0.198	0.990	0.199	N/A
	StrHunter	0.509	0.184	0.271	0.978	0.298	0.942
	CRHunter	0.451	0.214	0.290	0.982	0.303	0.946
High quality model	StrFeature	0.504	0.166	0.250	0.976	0.280	0.931
	StrTemplate	0.166	0.279	0.208	0.990	0.210	N/A
	StrHunter	0.486	0.163	0.244	0.976	0.272	0.932
	CRHunter	0.446	0.217	0.292	0.983	0.303	0.942
Low quality model	StrFeature	0.427	0.140	0.211	0.974	0.234	0.916
	StrTemplate	0.129	0.223	0.164	0.990	0.165	N/A
	StrHunter	0.417	0.140	0.209	0.975	0.231	0.918
	CRHunter	0.430	0.211	0.283	0.983	0.294	0.937

[a] High and low quality models denote the structures modelled by I-TASSER with relaxed and strict sequence identity cutoffs (90% and 30%), respectively.
[b] Annotations of different methods are the same as those in Table 1.

Table S3. Comparison with other prediction methods

Method[a]	Reported measure[b]	EF family	EF superfamily	EF fold	HA superfamily	PC	T124
CRpred[c]	Recall(Precision)	0.583(0.186)	0.521(0.170)	0.482(0.170)	0.540(0.149)	0.537(0.175)	0.501(0.147)
CatANalyst[d]	Recall(Precision)	0.613(0.205)	0.662(0.239)	0.646(0.241)	0.674(0.210)	0.697(0.225)	0.548(0.155)
SeqHunter	Recall(Precision)	0.612(0.207)	0.514(0.168)	0.431(0.160)	0.642(0.178)	0.360(0.143)	0.565(0.185)
CRHunter	Recall(Precision)	0.710(0.262)	0.686(0.219)	0.627(0.213)	0.732(0.242)	0.668(0.221)	0.715(0.286)

[a] SeqHunter is the combination of our sequence-based feature and template methods. CRHunter is our final prediction algorithm.
[b] Recall (precision) values of CatANalyst, SeqHunter, and CRHunter are reported when their precision (recall) values are equal to those of CRpred.
[c] Results on the above six datasets reported from Zhang *et al.*[23].
[d] Results on the above six datasets reported from Cilia and Passerini[27].

References

1. Hubbard, S. J. & Thornton, J. M. "NACCESS", Computer Program. *Department of Biochemistry and Molecular Biology, University College London* (1993).
2. Mihel, J., Sikić, M., Tomić, S., Jeren, B. & Vlahovicek, K. PSAIA - protein structure and interaction analyzer. *BMC Struct. Biol.* **8**, 21 (2008).
3. Le Guilloux, V., Schmidtke, P. & Tuffery, P. Fpocket: an open source platform for ligand pocket detection. *BMC Bioinformatics* **10**, 168 (2009).
4. Kabsch, W. & Sander, C. Dictionary of protein secondary structure: pattern recognition of hydrogen-bonded and geometrical features. *Biopolymers* **22**, 2577-2637 (1983).
5. McDonald, I. K. & Thornton, J. M. Satisfying hydrogen bonding potential in proteins. *J. Mol. Biol.* **238**, 777-793 (1994).
6. Altschul, S. F. *et al.* Gapped BLAST and PSI-BLAST: a new generation of protein database search programs. *Nucleic Acids Res.* **25**, 3389-3402 (1997).
7. Capra, J. A. & Singh, M. Predicting functionally important residues from sequence conservation. *Bioinformatics* **23**, 1875-1882 (2007).
8. Shenkin, P. S., Erman, B. & Mastrandrea, L. D. Information-theoretical entropy as a measure of sequence variability. *Proteins* **11**, 297-313 (1991).
9. Mirny, L. A. & Shakhnovich, E. I. Universally conserved positions in protein folds: reading evolutionary signals about stability, folding kinetics and function. *J. Mol. Biol.* **291**, 177-196 (1999).
10. Williamson, R. M. Information theory analysis of the relationship between primary sequence structure and ligand recognition among a class of facilitated transporters. *J. Theor. Biol.* **174**, 179-188 (1995).
11. Caffrey, D. R., Somaroo, S., Hughes, J. D., Mintseris, J. & Huang, E. S. Are protein-protein interfaces more conserved in sequence than the rest of the protein surface? *Protein Sci.* **13**, 190-202 (2004).
12. Wang, K. & Samudrala, R. Incorporating background frequency improves entropy-based residue conservation measures. *BMC Bioinformatics* **7**, 385 (2006).
13. Faraggi, E., Zhang, T., Yang, Y., Kurgan, L. & Zhou, Y. SPINE X: improving protein secondary structure prediction by multistep learning coupled with prediction of solvent accessible surface area and backbone torsion angles. *J. Comput. Chem.* **33**, 259-267 (2012).
14. Zhang, T. *et al.* SPINE-D: Accurate prediction of short and long disordered regions by a single neural-network based method. *J. Biomol. Struct. Dyn.* **29**, 799-813 (2012).
15. Kawashima, S. *et al.* AAindex: amino acid index database, progress report 2008. *Nucleic Acids Res.* **36**, 5 (2008).
16. Chen, K., Mizianty, M. J. & Kurgan, L. Prediction and analysis of nucleotide-binding residues using sequence and sequence-derived structural descriptors. *Bioinformatics* **28**, 331-341 (2012).
17. Han, L., Zhang, Y.-J., Song, J., Liu, M. S. & Zhang, Z. Identification of catalytic residues using a novel feature that integrates the microenvironment and geometrical location properties of residues. *PLoS One* **7**, e41370 (2012).
18. Andreeva, A. *et al.* SCOP database in 2004: refinements integrate structure and sequence family data. *Nucleic Acids Res.* **32**, 9 (2004).
19. Youn, E., Peters, B., Radivojac, P. & Mooney, S. D. Evaluation of features for catalytic residue prediction in novel folds. *Protein Sci.* **16**, 216-226 (2007).
20. Chea, E. & Livesay, D. R. How accurate and statistically robust are catalytic site predictions based on closeness centrality? *BMC Bioinformatics* **8**, 153 (2007).
21. Gutteridge, A., Bartlett, G. J. & Thornton, J. M. Using a neural network and spatial clustering to predict the

location of active sites in enzymes. *J. Mol. Biol.* **330**, 719-734 (2003).
22. Petrova, N. V. & Wu, C. H. Prediction of catalytic residues using Support Vector Machine with selected protein sequence and structural properties. *BMC Bioinformatics* **7**, 312 (2006).
23. Zhang, T. *et al.* Accurate sequence-based prediction of catalytic residues. *Bioinformatics* **24**, 2329-2338 (2008).
24. Yang, J. *et al.* The I-TASSER Suite: protein structure and function prediction. *Nat. Methods* **12**, 7-8 (2015).
25. Fu, L., Niu, B., Zhu, Z., Wu, S. & Li, W. CD-HIT: accelerated for clustering the next-generation sequencing data. *Bioinformatics* **28**, 3150-3152 (2012).
26. Li, S., Yamashita, K., Amada, K. M. & Standley, D. M. Quantifying sequence and structural features of protein-RNA interactions. *Nucleic Acids Res.* **42**, 10086-10098 (2014).
27. Cilia, E. & Passerini, A. Automatic prediction of catalytic residues by modeling residue structural neighborhood. *BMC Bioinformatics* **11**, 115 (2010).
